# A Review of the Efficacy of Ultraviolet C Irradiation for Decontamination of Pathogenic and Spoilage Microorganisms in Fruit Juices

**DOI:** 10.4014/jmb.2212.12022

**Published:** 2023-01-20

**Authors:** Ahmad Rois Mansur, Hyun Sung Lee, Chang Joo Lee

**Affiliations:** 1 Enterprise Solution Research Center, Korea Food Research Institute, Wanju 55365, Republic of Korea; 2 Department of Food Science and Biotechnology, Wonkwang University, Iksan, Jeonbuk 54538, Republic of Korea

**Keywords:** Ultraviolet C irradiation, fruit juices, microbial decontamination, food safety

## Abstract

Ultraviolet C (UV-C, 200–280 nm) light has germicidal properties that inactivate a wide range of pathogenic and spoilage microorganisms. UV-C has been extensively studied as an alternative to thermal decontamination of fruit juices. Recent studies suggest that the efficacy of UV-C irradiation in reducing microorganisms in fruit juices is greatly dependent on the characteristics of the target microorganisms, juice matrices, and parameters of the UV-C treatment procedure, such as equipment and processing. Based on evidence from recent studies, this review describes how the characteristics of target microorganisms (*e.g.*, type of microorganism/strain, acid adaptation, physiological states, single/composite inoculum, spore, etc.) and fruit juice matrices (*e.g.*, UV absorbance, UV transmittance, turbidity, soluble solid content, pH, color, etc.) affect the efficacy of UV-C. We also discuss the influences on UV-C treatment efficacy of parameters, including UV-C light source, reactor conditions (*e.g.*, continuous/batch, size, thickness, volume, diameter, outer case, configuration/arrangement), pumping/flow system conditions (*e.g.*, sample flow rate and pattern, sample residence time, number of cycles), homogenization conditions (*e.g.*, continuous flow/recirculation, stirring, mixing), and cleaning capability of the reactor. The collective facts indicate the immense potential of UV-C irradiation in the fruit juice industry. Existing drawbacks need to be addressed in future studies before the technique is applicable at the industrial scale.

## Introduction

The global consumption of fruit beverages/juices has been steadily increasing, reaching 95.69 billion liters in 2018 [1]. High consumption of fruit juices may be influenced by consumers’ perception that fruit juices are healthy foods that are enriched in vitamins, minerals, and dietary fibers and are also rich in health-associated bioactive compounds [2, 3]. However, fruit juices are highly perishable and can be easily contaminated by pathogenic and spoilage microorganisms. Several bacterial pathogens, including *Escherichia coli* O157:H7, *Listeria monocytogenes*, *Salmonella* spp., *Shigella* spp., and *Staphylococcus aureus*, have been associated with foodborne illnesses in fruit juices. Lactic acid bacteria and spore forming Alicyclobacillus spp. have been associated with spoilage in fruit juices [4]. Bacterial spores are not a direct hazard to the food industry. However, their eventual germination, outgrowth, and proliferation may cause foodborne illnesses and spoilage [5]. Yeast growth in fruit juices may result in undesirable metabolic products, such as carbon dioxide and alcohol. Yeast enzymes can also cause a number of problems in fruit juices that include enhanced turbidity, flocculation, and phase separation. Similar to yeast, mold growth produces undesirable effects, such as gas production, odor, and formation of a mycelial mat on the juice surface [6]. Furthermore, molds may produce spores that are highly heat-resistant and can survive pasteurization [7]. Therefore, treatments that can ensure the safety and quality of fruit juices are necessary.

Traditional thermal techniques have been commonly used to decontaminate fruit juices, extend their shelf life, and maximize the performance of fruit juice processing. However, such techniques may detrimentally affect nutritional, physicochemical, rheological, and organoleptic properties of fruit juices [8, 9]. To overcome these effects, numerous nonthermal alternatives, including UV-C irradiation, have been developed to ensure the safety and quality of fruit juices [10].

UV-C light irradiation is permitted in the United States as a decontamination method for fruit juices [11, 12]. Short wavelength UV-C light (200–280 nm) has germicidal potential, in which UV-C light can penetrate the cell wall of microorganisms, disrupt their DNA, alter their metabolism and reproduction, and lead to cell death [13]. Several recent reviews have summarized the applications of UV-C technology using continuous or batch reactors in the processing of liquid foods, including fruit juices. The efficacy of UV-C irradiation in the reduction of microorganisms in fruit juices is dependent on several factors, including the characteristics of target microorganisms, juice matrices, and parameters of UV-C equipment and processing [14–16].

Based on recent evidence from studies from 2017 to 2022, the present review mainly aims to discuss how the aforementioned factors influence the efficacy of UV-C irradiation in reducing specific microorganisms in fruit juices. In addition, considering that any changes in quality attributes after UV-C treatment need to be evaluated when developing and applying this technique, the effects of this technique on the quality of fruit juices are also briefly discussed.

## Efficacy of UV-C Varies depending on Characteristics of Target Microorganisms

The sensitivity and resistance of microorganisms to UV irradiation varies greatly. This variation is mainly due to the differences in cellular components, such as structure, thickness, and composition of the cell wall, structure and type of cellular proteins/nucleic acid, physiological state, photoproducts, and cell ability to repair UV damage [17]. The results from published studies (Table 1) highlight the marked variation in sensitivity to UV-C irradiation among microbial group/species. For example, *E. coli* was found to be more resistant to UV-C treatment than *S. enterica* and *L. monocytogenes* when two different UV-C batch reactors (maximum dose up to 3.64 or 6.00 J/cm^2^) were applied to decontaminate apple juices [18]. *E. coli* was also slightly more resistant than S. Typhimurium in cranberry flavored water treated with continuous UV-C. The authors reported that a 5 log reduction of *E. coli* and S. Typhimurium required UV-C treatment at a dose of 21 and 20 mJ/cm^2^, respectively [19]. In contrast, *E. coli* was less resistant than *L. monocytogenes* in coconut water treated with continuous UV-C (total doses of 0.43–0.80 J/ml)[20]. Compared to *Lactobacillus plantarum* and *Pseudomonas fluorescens*, *E. coli* was also less resistant to UV-C irradiation. For instance, higher log reductions of *E. coli* than those of *L. plantarum* were reported in two blended juices [orange-tangerine (OT) and orange-banana-kiwi-mango-strawberry (OBKMS)] exposed to continuous UV-C in a pilot-scale system (dose of 1,670 J/L for 31 cycles; maximum dose of 0.39 J/cm^2^) [21, 22] and those of *P. fluorescens* in pomegranate juice treated with a continuous UV-C (dose of 2.12 J/ml for three cycles) [23]. Variations in the sensitivity to the UV-C irradiation were also observed among 17 strains of yeast in orange juices, in which a nearly 5-log reduction required UV-C irradiation (batch reactor) at doses of 0.6–7.2 J/cm^2^ [24].

Regardless of other factors, such as variations in juice matrices and UV-C treatment procedure, the UV resistance of yeasts in fruit juices seems higher than that of bacteria (Table 1). For instance, continuous UV-C treatment (dose of 2.12 J/ml for three cycles) of pomegranate juice did not have any major impact on *Saccharomyces cerevisiae*, with less than one log reduction attained. However, *E. coli* exhibited greater reduction to the same UV-C treatment, in which approximately a 4.38log reduction was achieved [23]. Similar trends were also reported by Fenoglio *et al*. [21] and Ferrario *et al*. [22] who investigated the efficacy of a pilot-scale continuous UV-C (maximum dose of 0.39 J/cm^2^) on the reduction of microbial populations in the blended OBKMS and OT juices. *S. cerevisiae* appeared to be more resistant than *L. plantarum* and *E. coli* when exposed to UV-C treatment in all tested juice matrices [21, 22].

The results from previous studies (Table 1) also reveal that the characteristics of the microbial inoculum, including acid adaptation, physiological states, single/composite, and presence of spores, influence the efficacy of UV-C irradiation. For instance, the length of exposure time of apple juice to 0.17 mW/cm^2^ UV-C in a batch reactor needed to achieve a 5 log reduction in acid adapted *E. coli* O157:H7 and S. Typhimurium were significantly longer than those for non-acid adapted cells. This might be due to changes in the cellular fatty acid composition following acid adaptation, which induced the enhanced resistance of both pathogens to UV-C treatments [25]. Comparison of the responses of S. Typhimurium at three physiological states (exponential, stationary, and long-term survival [LTS] phases) in apple juice following UV-C irradiation (1.5 mW/cm^2^ for 0–12 min in a batch reactor) revealed that the LTS cells were more resistant to UV-C than the exponential and stationary cells. After 4–12min of UV-C treatment in apple juice, regardless of juice pH, log reductions were significantly lower for LTS cells, indicating that LTS cells were far less impacted by UV-C irradiation than stationary phase cells followed by exponential phase cells [26].

Because inter-strain variations in sensitivity to UV-C irradiation have been reported [27], one study used four strains as representatives for each target microorganism to permit a more adequate assessment [18]. Moreover, an assessment of the UV-C resistance of 17 yeast strains (inter- and intra-strains) in composite cultures demonstrated that the composite cultured yeasts were more resistant than single cultures. A reduction of close to 5 logs for a composite culture of yeasts in orange juice required UV-C irradiation at doses of 6.4–7.2 J/cm^2^ in a batch reactor, compared to doses ranging from 0.6 to 3.6 J/cm^2^ for single culture of each yeast strain depending on the type or strain [24]. Reductions of single strains of *S. cerevisiae* exceeding 4 logs were achieved in OT juice blend continuously treated with 1.72 J/cm^2^ UV-C. The findings were not significantly different from those of mixed inocula of *S. cerevisiae* and *E. coli* or native microflora comprised of native yeasts, molds, and mesophilic aerobic bacteria. These results suggest that the native microflora in the juice sample did not affect the inactivation of *S. cerevisiae* by UV-C. However, reductions of less than 3 logs for the total yeast population were obtained when *S. cerevisiae* was composited with three other yeast strains (*Pichia anomala*, *Zygosaccharomyces bailii*, and *Candida parapsilosis*). The lower effectiveness of UV-C in inactivating *S. cerevisiae* in the presence of other yeast strains might be attributed to the large size of yeast cells; the large cells might interfere with the light path and impair the inactivation process [28].

Microbial spores are more resistant to UV-C than vegetative cells [29]. In one study, a 15-min batch UV-C treatment (12.6 kJ/m^2^) of *A. acidoterrestris* spores in orange juice led to a reduction of less than 2 log colony forming units (CFU)/ml [30]. In another study, batch UV-C treatments (11.50 or 13.44 W/m^2^ for 8 min, and 0.34 W/m^2^ for 25 min) produced 5 log reductions of *A. acidoterrestris* spores in apple juice [31]. For the inactivation of fungal spores, application of a batch UV-C treatment (36 W/m^2^) for 10 min allowed the 5.7 log reduction of *Aspergillus fischeri* and 4.2 log reduction of *Paecilomyces niveus* in apple juice. When batch UV-C irradiation was applied at a much lower intensity of 6.5 W/m^2^, such reductions were not achieved, even after 30 min of exposure [32]. Inactivation of ascospores of *Talaromyces macrosporus* and *Neosartorya spinosa* in apple juice treated with batch UV-C treatments (doses of 7.2, 14.3, or 21.5 J/ml for 1-3 cycles) was reported. However, most of the tested UV-C treatments were not sufficient to attain 5 log reductions of the ascospores, except for *N. spinosa* ascospores treated with 21.5 J/ml UV-C [33].

## Efficacy of UV-C Varies depending on Juice Matrices

Optical and physical characteristics of the treated food matrix, such as UV absorbance (UVA), UV transmittance (UVT), turbidity (nephelometric turbidity units, NTU), soluble solids content (°Brix), pH, and color, influence the efficacy of UV-C treatment. Generally, UV-C efficacy will be lower for a more complex food matrix (*e.g.*, juices that are turbid, colored, and/or suspended solids) [16, 29, 34]. Evidence from recent studies (Table 1) clearly shows that the juice matrix has a remarkable effect on UV-C efficacy. Thus, the use of the same UV-C treatment for the decontamination of different types of juices may result in different reductions of specific target microorganisms. For instance, a pilot-scale continuous UV-C treatment (0.39 J/cm^2^) was highly effective for decontamination of clear pear juice (UVT 89.1%, UVA 0.05/cm, 21.9 NTU), achieving up to 4.4 and 5.5 log reductions for *L. plantarum* and *E. coli*, respectively [21]. However, a much lower efficacy of the same UV-C treatment was observed after decontamination of turbid juice blends, yielding less than 4 log reductions of both bacteria in both blended OT (UVT 20.9%, UVA 0.68/cm, 3,100 NTU) and OBMKS (UVT 42.6%, UVA 0.37/cm, 1,767 NTU) juices. Overall, the results of this study suggest that the higher values of UVT and lower values of UV absorbance, turbidity, and color parameter (a*) of the juice samples are closely associated with the higher efficacy of the tested UV-C treatment [21]. Turbid juices with suspended solids have a higher UV absorbance than clear juices. An increase in the level of UV absorbance leads to a decrease in the ability of UV-C light to penetrate food, thereby reducing the antimicrobial efficiency of any UV-C dose [35]. Moreover, the presence of suspended solids and soluble components in the food matrix can weaken the effects of UV-C irradiation by inducing light scattering, absorption, and reflection [15]. In addition, differences in the pH of juice samples are also likely to affect the efficacy of UV-C applied at 1.5 mW/cm^2^ for up to 12 min in a batch reactor, in which *S*. Typhimurium cells from all three growth phases exhibited less reduction in apple juice with an adjusted pH of 5.65, compared to apple juice with a lower pH of 3.63 [26]. Therefore, the development and application of UV-C irradiation for microbial inactivation should encompass a broad range of juice products.

## Efficacy of UV-C Varies depending on UV-C Decontamination Procedure

The comparison of UV-C decontamination procedures from different studies is challenging because the results have varied in terms of scale (*e.g.*, pilot-scale vs lab/small scale) and equipment and processing parameters (see Table 1 for more details). Moreover, most studies only reported the applied and/or incident UV-C doses/intensities, even though the absorbed dose (the irradiant energy absorbed by the food components available for driving the solution reaction) and delivered dose (the actual amount of irradiant energy delivered to the microorganism) are more critical for enhanced microbial inactivation [29]. In general, the results from most of the studies (Table 1) reveal that higher UV-C doses/intensities led to higher rates of microbial inactivation, regardless of the target microorganism and juice sample characteristics. To ensure that the dose/intensity from the UV-C light source is reliably delivered to the irradiated product and enhances the antimicrobial efficiency, a specially designed UV-C continuous or batch reactor with different equipment and processing parameters is necessary. Numerous UV-C continuous reactors, including curved and coiled tubes, and batch reactors, including petri dish, well plate, and tank, with different arrangements and configurations have been developed and applied for the decontamination of fruit juices (Table 1).

The main part of the UV-C reactor is the UV-C lamp/light source. The target microorganisms in the juice samples are exposed to UV-C light at a certain intensity/irradiance for a certain time. Therefore, selecting a UV-C lamp with appropriate features for microbial inactivation, which include wavelength, power, and size, is an important step to enhance the penetration of UV-C light into juice samples. The efficiency of continuous and batch UV-C decontamination procedures for microbial inactivation can be greatly affected by the UV-C lamp features (*e.g.*, type, number, and position of the lamp and its outer case/layer) and irradiation time. In recent years, different types of UV-C germicidal lamps with various wavelengths ranging from 200 to 280 nm, such as krypton-chlorine excimer lamps (222 nm), general UV-C lamps (253–254 nm), and light emitting diode lamps (254–279 nm), have been studied for their antimicrobial efficacy (Table 1). The effects of using one or two UV-C lamps (30 or 80 cm length) connected in series on the reduction of *S. cerevisiae* in grape juice have been reported. No significant difference in the obtained log reductions (approximately 2 log CFU/ml) was observed between the 30 and 80 cm UV-C lamp. However, by increasing the number of tested UV-C lamps, the inactivation efficacy was enhanced (>5 log CFU/ml) because the UV-C intensity was doubled [36]. The results summarized in Table 1 also show that an increase in the irradiation time would increase the UV-C dose/intensity, allowing greater reductions in the target microorganisms in fruit juice samples [19, 25, 26, 28, 33, 37-40]. However, in some cases, further irradiation up to certain exposure times did not cause any significant increase in the microbial reduction [24, 32]. Furthermore, several equipment and processing parameters applied in the UV-C decontamination procedure (Table 1), such as reaction tube/tank/plate (*e.g.*, size, thickness, volume, diameter, outer case, configuration/arrangement), pumping/flow system (*e.g.*, sample flow rate and pattern, sample residence time, number of cycles), homogenization (*e.g.*, continuous flow/recirculation, stirring, mixing), and cleaning capability of the reactor, may also influence the efficacy of the UV-C decontamination process. All of these parameters need to be evaluated during the development and optimization of UV-C decontamination procedures.

UV-C light treatment is more frequently used for surface sterilization because it does not penetrate food samples very deeply. One of the strategies that can be applied for juice treatment is the use of a thin layer to increase the surface area and decrease the depth of the product [13]. For instance, using a reaction tube with a lower thickness (0.5 cm) led to enhanced reductions in *S. cerevisiae* [36]. Reducing the thickness of the juice film was highly effective, because greater reductions in *S. cerevisiae* were achieved at any of the tested flow rates, even when using a single UV-C lamp. The results may reflect the narrower distance between the juice sample and UV-C lamp in thinner reaction tubes, thus permitting better light penetration and yeast inactivation [36]. The higher log reductions of *E. coli* and *L. monocytogenes* in tender coconut water conveyed in a thinner reaction tube (inner diameter of 1.6 mm vs 3.2 mm) and treated with continuous UV-C irradiation were also reported [20]. However, reducing the thickness of the tube/juice film might cause laminar flow when the juice matrix passes through the reactor. Consequently, UV-C light mostly penetrates the outer layers of the flowing liquid, leaving the inner layers of the flowing liquid less exposed, which could restrict the delivery of a uniform dose/intensity into the sample [33].

To increase the probability of UV-C light penetrating the sample and delivering uniform dose/intensity, the juice solution in the UV-C batch reactor can be mixed using a magnetic stir bar [40] or agitation system for the solution treated in the tank [18]. In the UV-C continuous reactor, the proper mixing of juice solutions can be estimated using the Reynolds and Dean numbers, which indicate the presence of turbulence and secondary flow, respectively, inside the UV-C reactor for any liquid food [41]. The presence of turbulent and secondary flows reportedly allows better mixing of the juice matrix inside the continuous reactor and higher exposure to UV-C light [20, 33]. Moreover, to allow for additional mixing, the samples were recirculated or cycled multiple times through the system. Recirculation of the juice solution compensates for the lack of turbulent flow and increases the probability that more parts of the juice matrix can be exposed to UV-C light. Increasing number of cycles increase the greater of UV-C exposure, and consequently increases the effectiveness of the treatment even at the same dose [33]. Multiple cycles of UV-C irradiation were more effective than single cycle for the inactivation of bacteria, yeast, and mold in various fruit juices [21-23, 33].

Since the UV-C dose is directly proportional to the average residence time and inversely proportional to the flow rate [19], enhanced microbial inactivation might also be achieved by controlling the flow rate or increasing the residence time to increase the delivered UV-C dose. For instance, in one study a lower flow rate of the sample led to a higher residence time of the sample, greater delivered dose, and increased efficacy of continuous UV-C treatment on the reduction of *E. coli* and *S. cerevisiae* in pomegranate juice [23]. However, in another study, an increase in the tested flow rates (from 5.2 ml/s to 17.1 or 31 ml/s) reportedly increased the reduction of *S. cerevisiae* in grape juice continuously treated with UV-C for 60 min [36]. This discrepancy might be due to other processing factors, such as the features of reaction tubes (*e.g.*, size, thickness, volume, diameter, outer case, configuration/arrangement) and flow pattern (laminar or turbulent flow) that affect the uniformity of the dose delivered to the juice sample and target microorganisms.

To further enhance the antimicrobial efficiency of UV-C irradiation, a combination of UV-C irradiation and other preservation techniques has been investigated. For instance, UV-C irradiation combined with microwave heating [23], mild heat [21, 22, 25, 37, 39], encapsulated vanillin and citral emulsions [22], chitosan nanoparticles [42], ohmic heating [43], nisin [30], ultrasonic atomizer nozzles [44], and ultra-high-pressure homogenization [33] have been reported to exhibit synergistic and/or additive microbial inactivation effects.

Cleaning the UV-C reactor before and after use is necessary to maintain cleanliness and sterility. Several methods that have been used include cleaning with 500 ml of hot water (70°C) followed by 100 ml of hypochlorite (200 ppm) for 10 min, and sterile deionized water at room temperature for 4 min [20]. In other studies, cleaning by recirculating sterile water (~200ml/min) at room temperature for 3 min [23] or sequential flushing with sterile water, 0.1 N HCl, sterile water, 0.1 N NaOH, and a final rinse with sterile water [19] have also been performed.

## Effects of UV-C Irradiation on Quality of Fruit Juices

The effects of UVC irradiation on the quality attributes of fruit juices should be evaluated when developing and applying this treatment. As shown in Table 2, most studies have reported no significant changes in the physicochemical properties that include °Brix, viscosity, optical density, density, pH, acidity, turbidity, browning index, reducing sugar, and color of fruit juices after UV-C irradiation. However, in some cases, adverse effects of UV-C irradiation on the bioactive compounds, including phenolics, antioxidants, anthocyanin, and vitamin C, and color of the treated fruit juices were observed.

## Conclusions and Future Perspectives

Proper control of pathogenic and spoilage microorganisms during the processing of fruit juices is a prerequisite for ensuring food safety and maintaining hygiene standards. Based on evidence from recent studies, UV-C irradiation has shown immense potential in the fruit juice industry, and can be a good alternative to traditional thermal decontamination techniques. However, certain drawbacks restrict their industrial scale application. For instance, the microbial inactivation efficacy of UV-C irradiation is largely affected by the characteristics of target microorganisms and juice matrices. The low penetration of UV-C light demands higher doses and/or longer exposure times for the inactivation of highly resistant microorganisms, including yeasts, molds, and spore forming microorganisms, and decontamination of highly complex juice matrices (*e.g.*, turbid, large area/volume, colored). Such prolonged exposure to high UV-C doses may negatively impact the quality of juice, exceed the standards/permissible limits set by regulatory authorities, and may not be industrially feasible. Optimizing the UV-C treatment procedure, for example by using a thin layer reaction tube, may prevent light penetration losses, ensure a uniform delivered dose with shorter treatment time, and enhance the efficacy. However, it can be a handicap when scaling up to the industrial scale, in which the surface-to-depth ratios or flow rate should be maximized. Therefore, for UV-C irradiation technology to be accepted and effectively transferred to the juice industry, future studies should focus on process optimization/validation by taking into account the main factors (target microorganisms, juice matrices, and important parameters of UV-C treatment procedure) that affect treatment efficacy. The responses evaluated for such process optimization/validation should focus on microbial reduction and also on the quality (physicochemical, sensorial, and nutritional attributes) of fruit juices. A combination of UV-C irradiation with other food processing/preservation techniques can also be considered for enhanced efficiency and better outcomes. In addition, studies should be carefully performed at a lab/small scale before its realization at an industrial scale using the available/newly developed industrial scale UV-C decontamination device.

## Figures and Tables

**Table 1:** Efficacy of various ultraviolet C (UV-C) irradiation treatments on reduction of microorganisms in different fruit juices.

Target microorganism		Juice matrices		UV-C treatment procedure			Microbial reduction		Ref.

Type	Characteristics	Type	Characteristics ^a^	Equipment parameters ^b^	Processing parameters ^c^	Combined treatment ^d^	UV-C alone	Combined ^d^	
*Escherichia coli*	4 strains, initial load (5.7 log CFU/ml)	Apple juice	Commercial juice (pH 3.60, 11.2 °Brix, ρ 1045.0 g/L, UVT <0.01%); 1.2 ml sample (Chamber) and 14 L sample (Tank)	1). Batch reactor with chamber (61.8 × 27.7 × 20 cm), 3 lamps, and 12-well plates for sample (4 mm depth); 2). Batch reactor with 4 lamps inside a 15 L vertical tank (38 × 26 cm), peristaltic pump, air and water regulator	1). λ (254 nm), P (30 W); 30 min pre-irradiation, sample to lamp (12 cm), total dose (0.09-3.64 J/cm^2^); 2). λ (254 nm), P (17.2 W), 30 min preirradiation, total dose (0.60-6.00 J/cm^2^)	NA	<4 log CFU/ml	NA	[18]
	1 strain, initial load (6.0-7.0 log CFU/ml)	Pomegran ate juice	Commercial juice (pH 3.45, 11.33°Brix)	Continuous reactor with a 30 cm lamp and fluorinated ethylene propylene (FEP) coiled tubes (V: 98.96 ml) covered with stainlesssteel tube (4.75 cm ID × 5.1 cm OD)	λ (254 nm), P (14 W); 1 min pre-irradiation; flow rate (0.4 and 0.8 L/min); 3 cycles; dose per cycle (2.12 and 1.05 J/ml)	Microwave heating(1,000W, 2,450 MHz)	<5 log CFU/ml (UV-C 0.4 L/min for 3 cycles)	>6 log CFU/ml (UV-C 0.4 L/min for 3 cycles)	[23]
	1 strain, initial load (7.0-8.0 log CFU/ml)	Orange-tangerine juice	Juice blend (pH 3.5, ±10°Brix, UVT 20.9%, 3,100 NTU, UVA 0.68/cm)	Pilot scale continuous reactor with FEP coiled tubes (Length 13.9 m, ID 19 mm) with 12 lamps (4 inside, 8 outside) enclosed in a stainless-steel tube housing	λ (254 nm), P (input 432 W, output 176.4 W), flow rate (380 L/h), 31 cycles (each 36.6 s), dose per cycle (1,670 J/L), incident dose (0-0.39 J/cm^2^)	(1) Mild heat (50°C), and/or (2) Encapsulated vanillin (1,000 ppm) and citral (100) emulsions	UV-C 25°C (<5 log CFU/ml); UV-C 50 °C (>5 log CFU/ml)	UV-C 25°C (<5 log CFU/ml); UV-C 50°C (>5 log CFU/ml)	[21, 22]
	1 strain, initial load (7.0-8.0 log CFU/ml)	Orange-banana-kiwi-mango-strawberry juice	Juice blend (pH 3.7, ±10°Brix, UVT 42.6%, 1,767 NTU, UVA 0.37/cm)				UV-C 25°C (<5 log CFU/ml); UV-C 50 °C (>6 log CFU/ml)	UV-C 25°C (<5 log CFU/ml); UV-C 50°C (>6 log CFU/ml)	
	1 strain, initial load (7.0-8.0 log CFU/ml)	Pear juice	Commercial juice (pH 3.8, ±10°Brix, UVT 89.1%, 21.9 NTU, UVA 0.05/cm)			NA	UV-C 25°C (>5 log CFU/ml)	NA	[21]
	1 strain, initial load (7.0-8.0 log CFU/ml)	Grape juice	Commercial juice (pH 2.46, 15.5°Brix, 4.10 NTU); 15 ml sample	Batch reactor with 4 lamps, a glass slide (7.5 × 2.5 × 0.1 cm) coated with/out a 10 cm^2^ photocatalyst placed under a 100 × 15 mm petri dish (depth 0.24 cm), and magnetic stirrer	λ (254 nm), I (19.7 mW/cm^2^ 5-60 min)	TiO_2_-SiO_2_ photocatalyst	UV-C ≥20 min (5 log CFU/ml)	UV-C≥20min (5 log CFU/ml)	[40]
	1 strain, initial load (8.0 log CFU/ml)	Cranberry flavored water	Self-produced juice (pH 2.9, 8°Brix, UVT 0.004%, UVA 4.43/cm); 4 L sample	Continuous reactor with inlet and outlet tank, peristaltic pump, and 1 lamp and teflon curved tube for sample inside a tank with air flow	λ (254 nm), exposure time (115 and 403 s), total dose (6 and 21 mJ/cm^2^)	NA	UV-C 21 mJ/cm^2^ (>5 log CFU/ml)	NA	[19]
	1 strain, initial load (6.0-8.0 log CFU/ml)	Carrot-orange juice	Juice blend (pH 3.8; 10.6°Brix; 7,667 NTU; UVA 0.32/cm); 750 ml sample	Continuous reactor with 2 connected lamps inside a 0.87-mm glass tube (OD 0.031 m, ID 0.024 m, 0.22 L), inlet and outlet flexible hoses, double jacket vessel, thermostatic water bath (20, 40, 45, or 50°C), and a peristaltic pump	λ (253.7 nm), P (30 W), 15 min pre-irradiation, flow rate (1.6 L/min), exposure time (0-15 min), total dose (0-10.6 kJ/m^2^)	Mild heat (40-50°C)	UV-C 15 min (<3 log CFU/ml)	UV-C 50°C 15 min (>5 log CFU/ml)	[37]
	1 strain, initial load (8.0 log CFU/ml)	Coconut water	pH 5.09; 6.8°Brix; ρ 1,015.6 kg/m^3^; UVA 1.90/cm	Continuous reactor with a 60 cm lamp covered with quartz glass sleeve, coiled perfluoroalkoxy (PFA) tubes [1^st^ reactor (240-480 cm, 1.6 mm ID) and 2^nd^ reactor (120-240 cm, 3.2 mm ID)], and three valves	λ (254 nm), P (8.7 W), I [2.38 mW/cm^2^ (1.6 mm) and 3.79 mW/cm^2^ (3.2 mm)], RT (14 s), flow rate [20-60 ml/min (1.6 mm) and 40-120 ml/min (3.2 mm)], total dose [0.80 J/ml (1.6 mm) and 0.43 J/ml (3.2 mm)]	NA	>5 log CFU/ml [UV-C 60 ml/min (1.6 mm)] and <5 log CFU/ml [UV-C 120 ml/min (3.2 mm)]	NA	[20]
*E. coli* O157:H7	1 strain, initial load (6.0 log CFU/ml)	Pomegran ate juice	Not specified	Batch reactor with 1 lamp	λ (253 nm), 15 min preirradiation, dose [0.682 J/cm^2^ 45 min (individual) or 0.281 J/cm^2^ 20 min (combined)], sample to lamp (30 cm)	ChNPs (100 μL/ml)	±3.4 log CFU/ml	±4 log CFU/ml	[42]
	3 strains (non-acid or acid adapted cells), initial load (8.0 log CFU/ml)	Apple juice	Commercial juice (pH 2.24, 18°Brix, 17.6 NTU); 20 ml sample	Batch reactor with a DBD-driven KrCl excilamp (24 × 10 × 10 cm^3^) inside a rectangular metal case with a 10 × 4 cm^2^ window, and shaking water bath at 100 rev/min	λ (222 nm), P (110 W), I (0.17 mW/cm^2^ 5-11 min), sample to lamp (20 cm)	Mild heating (45-55°C)	<5 log CFU/ml (increased as treatment time increased)	±7logCFU/ml (increased as treatment temperature and time increased)	[25]
	3 strains, initial load (6.0-7.0 log CFU/ml)	Tomato juice	Pasteurized juice (pH 3.6, 11.8°Brix); 50 ml sample	Not specified	λ (254 nm), total dose (0.19 J/cm^2^)	Ohmic heating (63°C, 210 s),	<1 log CFU/ml	4-5 log CFU/ml	[43]
*E. coli* K12	1 strain, initial load (6.0-7.0 log CFU/ml)	Apple juice	Turbid/clear juice (pH 3.9, 13.5°Brix, 4.43-1,619 NTU, 5.91-28.53/cm); 3 ml sample	Batch reactor with 4 UV-LEDs (each 8.33 mm ID, λ at 254, 280, 365, and 405 nm) and magnetic stirrer	UV LED λ (254-405 nm), exposure time (20-40 min), sample in petri dish (0.15 cm depth) to lamp (1 cm)	UV-A and/or UV-B LED	UV-C 254 nm (<4 log CFU/ml clear juice, <2 log CFU/ml turbid juice)	Combined (<4 log CFU/ml clear juice, <2 log CFU/ml turbid juice)	[45]
*Salmonella enterica*	4 strains, initial load (5.70 log CFU/ml)	Apple juice	Commercial juice (pH 3.60, 11.2°Brix, ρ 1045.0 g/L, UVT <0.01%); 1.2 ml sample (Chamber) and 14 L sample (Tank)	1). Batch reactor with chamber (61.8 × 27.7 × 20 cm), 3 lamps, and 12-well plates for sample (4 mm depth); 2). Batch reactor with 4 lamps inside a 15 L vertical tank (38 × 26 cm), peristaltic pump, air and water regulator	1). λ (254 nm), P (30 W); 30 min pre-irradiation, sample to lamp (12 cm), total dose (0.09-3.64 J/cm^2^); 2). λ (254 nm), P (17.2 W), 30 min preirradiation, total dose (0.60-6.00 J/cm^2^)	NA	UV-C chamber 3.64 J/cm^2^ (>5 log CFU/ml); UV-C tank (<4 log CFU/ml)	NA	[18]
*S*. Typhimurium	3 strains (non-acid and acid adapted cells), initial load (8.0 log CFU/ml)	Apple juice	Commercial juice (pH 2.24, 18°Brix, 17.6 NTU); 20 ml sample	Batch reactor with a DBD-driven KrCl excilamp (24 × 10 × 10 cm^3^) inside a rectangular metal case with a 10 × 4 cm^2^ window, and shaking water bath at 100 rev/min	λ (222 nm), P (110 W), I (0.17 mW/cm^2^ 5-11 min), sample to lamp (20 cm)	Mild heating (45-55°C)	<3 log CFU/ml (increased as treatment time increased)	±7logCFU/ml (increased as treatment temperature and time increased)	[25]
	3 strains, initial load (6.0-7.0 log CFU/ml)	Tomato juice	Pasteurized juice (pH 3.6, 11.8°Brix); 50 ml sample	Not specified	λ (254 nm), total dose (0.19 J/cm^2^)	Ohmic heating (63°C, 210 s),	<1 log CFU/ml	2-3 log CFU/ml	[43]
	1 strain [exponential (E), stationary (S), and Longterm survival (LTS) cells)], initial load (7.0 log CFU/ml)	Apple juice	Sterilized juice (pH 3.63 or 5.65); 5 ml sample	Batch reactor with 1 lamp, 60 × 15 mm petri dish (depth 0.2 cm), and electrical stirrer (5 rpm)	λ (254 nm), 10 min preirradiation, I (1.5 mW/cm^2^ 0-12 min)	NA	UV-C 12 min pH 3.63: >5 log CFU/ml(Ecell), >4 log CFU/ml (S cell), <4 log CFU/ml (LTS cell); UV-C 12 min pH 5.65: ±4 log CFU/ml (E cell), >3 log CFU/ml (S cell), ±3 log CFU/ml (LTS cell)	NA	[26]
	1 strain, initial load (6.0 log CFU/ml)	Coconut water	pH 5.3, 4.4°Brix, UVA 1.25/cm; 2 L sample	Continuous reactor with receving tank, peristaltic pump, and 3 UVC lamps (quartz sleeve 29.3 × 2.3 cm) inside treatment tubes (stainless steel 29.5 × 4.8 cm)	λ (254 nm), P (12 W), I (0.044 W/cm^2^ per lamp, 3.5, 7, and 10.5 min), flow rate (1.9 ml/s), total dose (9.24, 18.48, and 27.72 J/cm^2^)	NA	UV-C 7 min (>5 log CFU/ml); UV-C 10.5 min (>6 log CFU/ml)	NA	[38]
	1 strain, initial load (8.0 log CFU/ml)	Cranberry flavored water	Self-produced juice (pH 2.9, 8°Brix, UVT 0.004%, UVA 4.43/cm); 4 L sample	Continuous reactor with inlet and outlet tank, peristaltic pump, and 1 lamp and teflon curved tube for sample inside a tank with air flow	λ (254 nm), exposure time (94-376 s), total dose (5-20 mJ/cm^2^)	NA	UV-C 20 mJ/cm^2^ (>5 log CFU/ml)	NA	[19]
*Listeria monocytogenes*	4 strains, initial load (5.70 log CFU/ml)	Apple juice	Commercial juice (pH 3.60, 11.2°Brix, ρ 1045.0 g/L, UVT <0.01%); 1.2 ml sample (Chamber) and 14 L sample (Tank)	1). Batch reactor with chamber (61.8 × 27.7 × 20 cm), 3 lamps, and 12-well plates for sample (4 mm depth); 2). Batch reactor with 4 lamps inside a 15 L vertical tank (38 × 26 cm), peristaltic pump, air and water regulator	1). λ (254 nm), P (30 W); 30 min pre-irradiation, sample to lamp (12 cm), total dose (0.09-3.64 J/cm^2^); 2). λ (254 nm), P (17.2 W), 30 min preirradiation, total dose (0.60-6.00 J/cm^2^)	NA	UV-C chamber 3.64 J/cm^2^ (±5 log CFU/ml); UV-C tank (≤4 log CFU/ml)	NA	[18]
	1 strain, initial load (6.0 log CFU/ml)	Pomegran ate juice	Not specified	Batch reactor with 1 lamp	λ (253 nm), 15 min preirradiation, dose [0.682 J/cm^2^ 45 min (individual) or 0.281 J/cm^2^ 20 min (combined)], sample to lamp (30 cm)	ChNPs (100 μL/ml)	±1.5 log CFU/ml	Complete reduction (>5 log CFU/ml)	[42]
	3 strains, initial load (5.0-6.0 log CFU/ml)	Tomato juice	Pasteurized juice (pH 3.6, 11.8°Brix); 50 ml sample	Not specified	λ (254 nm), total dose (0.19 J/cm^2^)	Ohmic heating (63°C, 210 s),	<1 log CFU/ml	±5 log CFU/ml	[43]
	1 strain, initial load (8.0 log CFU/ml)	Coconut water	pH 5.09; 6.8°Brix; ρ 1,015.6 kg/m^3^; UVA 1.90/cm	Continuous reactor with a 60 cm lamp covered with quartz glass sleeve, coiled PFA tubes [1^st^ reactor (240-480 cm, 1.6 mm ID) and 2^nd^ reactor (120-240 cm, 3.2 mm ID)], and three valves	λ (254 nm), P (8.7 W), I [2.38 mW/cm^2^ (1.6 mm) and 3.79 mW/cm^2^ (3.2 mm)], RT (14 s), flow rate [20-60 ml/min (1.6 mm) and 40-120 ml/min (3.2 mm)], total dose [0.80 J/ml (1.6 mm) and 0.43 J/ml (3.2 mm)]	NA	>4 log CFU/ml [UV-C 60 ml/min (1.6 mm)] and <3 log CFU/ml [UV-C 120 ml/min (3.2 mm)]	NA	[20]
*Lactobacillus plantarum*	1 strain, initial load (7.0-8.0 log CFU/ml)	Orange-tangerine juice	Juice blend (pH 3.5, ±10°Brix, UVT 20.9%, 3,100 NTU, UVA 0.68/cm)	Pilot scale continuous reactor with FEP coiled tubes (Length 13.9 m, ID 19 mm) with 12 lamps (4 inside, 8 outside) enclosed in a stainless-steel tube housing	λ (254 nm), P (input 432 W, output 176.4 W), flow rate (380 L/h), 31 cycles (each 36.6 s), dose per cycle (1,670 J/L), incident dose (0-0.39 J/cm^2^)	(1) Mild heat (50°C), and/or (2) Encapsulated vanillin (1,000 ppm) and citral (100) emulsions	UV-C 25°C (<3 log CFU/ml); UV-C 50°C (>5 log CFU/ml)	UV-C 25°C (<3 log CFU/ml); UV-C 50°C (>5 log CFU/ml)	[21, 22]
	1 strain, initial load (7.0-8.0 log CFU/ml)	Orange-banana-kiwi-mango-strawberry juice	Juice blend (pH 3.7, ±10°Brix, UVT 42.6%, 1,767 NTU, UVA 0.37/cm)				UV-C 25°C (<4 log CFU/ml); UV-C 50°C (>6 log CFU/ml)	UV-C 25°C (<4 log CFU/ml); UV-C 50°C (>6 log CFU/ml)	
	1 strain, initial load (7.0-8.0 log CFU/ml)	Pear juice	Commercial juice (pH 3.8, ±10°Brix, UVT 89.1%, 21.9 NTU, UVA 0.05/cm)			NA	UV-C 25°C (<5 log CFU/ml)	NA	[21]
*Alicyclobacillus acidoterrestris*	Spores from 1 strain, initial load (4.0-5.0 log CFU/ml)	Orange juice	Commercial juice (pH 4, 11°Brix); 1 ml sample	Batch reactor with chamber (75 × 70 × 45 cm^3^), 3 lamps, 24-well cell culture plates for sample, and agitator	λ (254 nm), I (14 W/m^2^ for 3-15 min), 30 min preirradiation, total dose (2.52-12.6 kJ/m^2^), sample to lamp (24 cm)	Nisin (7.81 or 15.62 μg/ml)	UV-C 12.6 kJ/m^2^ (<2 log CFU/ml)	UV-C >2.52 kJ/m^2^ [complete reduction (LOD <1.7 log CFU/ml)]	[30]
	Spores from 1 strain, initial load (7.0 log CFU/ml)	Apple juice	Commercial juice (pH 3.2, 10.5°Brix, UVT 58%); 25 ml sample	Batch reactor with chamber (75 × 70 × 45 cm^3^), 3 lamps, 90-mm petridish for sample, and magnetic stirrer	λ (254 nm), P (15 W), 30 min pre-radiation, I (0.34-13.44 W/m^2^ for 0-25 min), sample in petridish (4 mm thickness) to lamp (30 cm)	NA	>5 log CFU/ml (UV-C 0.34-13.44 W/m^2^ for 25-8 min)	NA	[31]
*Pseudomonas fluorescens*	1 strain, initial load (6.0-7.0 log CFU/ml)	Carrot-orange juice	Juice blend (pH 3.8; 10.6°Brix; 7,667 NTU; UVA 0.32/cm); 750 ml sample	Continuous reactor with 2 connected lamps inside a 0.87-mm glass tube (OD 0.031 m, ID 0.024 m, 0.22 L), inlet and outlet flexible hoses, double jacket vessel, thermostatic water bath (20, 40, 45, or 50°C), and a peristaltic pump	λ (253.7 nm), P (30 W), 15 min pre-irradiation, flow rate (1.6 L/min), exposure time (0-15 min), total dose (0-10.6 kJ/m^2^)	Mild heat (40-50°C)	UV-C 15 min (<3 log CFU/ml)	UV-C 50°C 15 min (>5 log CFU/ml)	[37]
*Saccharomyces cerevisiae*	1 strain, initial load (8.0-9.0 log CFU/ml)	Orange juice	Pasteurized commercial juice (pH 3.48, 10.5°Brix); 20 ml sample	Batch reactor with chamber, 60 LED lamps (10 mm between lamp), tray and 90 mm petridish for sample with stirrer	UV-C LED λ (279 nm), total dose (0.16-1.42 J/cm^2^)	NA	UV-C LED 1.42 J/cm^2^ (4.44 log CFU/ml)	NA	[46]
	1 strain, initial load (6.0-7.0 log CFU/ml)	Pomegran ate juice	Commercial juice (pH 3.45, 11.33°Brix)	Continuous reactor with a 30 cm lamp and fluorinated ethylene propylene (FEP) coiled tubes (V: 98.96 ml) covered with stainlesssteel tube (4.75 cm ID × 5.1 cm OD)	λ (254 nm), P (14 W); 1 min pre-irradiation; flow rate (0.4 and 0.8 L/min); 3 cycles; dose per cycle (2.12 and 1.05 J/ml)	Microwave heating(1,000W, 2,450 MHz)	<1 log CFU/ml	>6 log CFU/ml (UV-C 0.4 L/min for 3 cycles)	[23]
	1 strain, initial load (6.0-7.0 log CFU/ml)	Orange-tangerine juice	Juice blend (pH 3.5, ±10°Brix, UVT 20.9%, 3,100 NTU, UVA 0.68/cm)	Pilot scale continuous reactor with FEP coiled tubes (Length 13.9 m, ID 19 mm) with 12 lamps (4 inside, 8 outside) enclosed in a stainless-steel tube housing	λ (254 nm), P (input 432 W, output 176.4 W), flow rate (380 L/h), 31 cycles (each 36.6 s), dose per cycle (1,670 J/L), incident dose (0-0.39 J/cm^2^)	(1) Mild heat (50°C), and/or (2) Encapsulated vanillin (1,000 ppm) and citral (100) emulsions	UV-C 25°C (<2 log CFU/ml); UV-C 50 °C (<5 log CFU/ml)	UV-C 25°C (<2 log CFU/ml); UV-C 50°C (<5 log CFU/ml)	[21, 22]
	1 strain, initial load (6.0-7.0 log CFU/ml)	Orange-banana-kiwi-mango-strawberry juice	Juice blend (pH 3.7, ±10°Brix, UVT 42.6%, 1,767 NTU, UVA 0.37/cm)				UV-C 25°C (<2 log CFU/ml); UV-C 50°C (<5 log CFU/ml)	UV-C 25°C (<2 log CFU/ml); UV-C 50°C (<5 log CFU/ml)	
	Single inoculum (1 strain); Composited inoculum with 3 yeast strains (*Pichia anomala*, *Zygo saccharomyces bailii*, *Candida parapsilosis*), 4 *E. coli* strains, or isolated native flora, initial load (5.0 log CFU/ml)	Orange-tangerine juice	Juice blend (pH 4.1, ±9°Brix, UVT 40.7%, 2,095 NTU); 750 ml sample	Continuous reactor with 2 connected lamps inside a 0.87-mm glass tube (OD 0.031 m, ID 0.024 m, 0.26 L), inlet and outlet flexible hoses, double jacket vessel, thermostatic water bath (20°C), and a peristaltic pump	λ (253.7 nm), exposure time (0-15 min), flow rate (1.6 L/min), total dose (0-1.72 J/cm^2^)	NA	>4 log CFU/ml (single inoculum or composited inoculum with *E. coli* or native flora), <4 log CFU/ml (composited inoculum with 3 yeast strains)	NA	[28]
	2 strains, initial load (4.0-5.0 log CFU/ml)	Orange juice	Commercial juice (pH 3.71, 11.60°Brix, insoluble solids 2.46%); 4 ml sample	Batch reactor with chamber, 3 lamps, and 35-mm petridish for sample with stirrer (1,500 rpm)	λ (254 nm), P (15 W), I (4.45 mW/cm^2^), dose (4.45 mJ/cm^2^ per s), exposure time (0-1,300 s), sample to lamp (10 cm)	NA	UV-C 0.6-2.0 J/cm^2^ (<5 log CFU/ml)	NA	[24]
	1 strain, initial load (6.0 log CFU/ml)	Grape juice	Commercial juice (pH 3.46, 13.10°Brix, ρ 1,057 kg/m^3^); 4 ml sample	Continuous reactor with 1 or 2 lamps (each 30 or 80 cm) inside stainless steel tube (ID 4.8 cm; thickness of the gap: 1.0 or 0.5 cm), peristaltic pump, and cylindrical vessel connected to a water-bath (15°C)	λ (254 nm), P [(17 W (30 cm) or 38 W (80 cm)], 15 min pre-irradiation, flow rate (5.2, 17.1, 31 ml/s), I (10-11 mW/cm^2^ for 60 min)	NA	>5 log CFU/ml: [UV-C 2 lamps (30 or 80 cm), 1 cm thickness, 31 ml/s, 60 min, total dose 38-40 J/cm^2^)] or [UVC 1 lamp (30 cm), 0.5 cm thickness, 17 or 31 ml/s, 60 min, total dose 38 J/cm^2^)]	NA	[36]
	1 strain, initial load (5.0-6.0 log CFU/ml)	Carrot-orange juice	Juice blend (pH 3.8; 9.8-10.6°Brix; 707-7,667 NTU; UVA 0.17-0.32/cm); 750 ml sample	Continuous reactor with 2 connected lamps inside a 0.87-mm glass tube (OD 0.031 m, ID 0.024 m, 0.22 L), inlet and outlet flexible hoses, double jacket vessel, thermostatic water bath (20, 40, 45, or 50°C), and a peristaltic pump	λ (253.7 nm), P (30 W), 15 min pre-irradiation, flow rate (1.6 L/min), exposure time (0-15 min), total dose (0-10.6 kJ/m^2^)	Mild heat (50°C)	UV-C 15 min (<3 to <4 log CFU/ml)	UV-C 50°C 15 min (<4 to <5 log CFU/ml)	[37, 39]
	1 strain, initial load (6.0 log CFU/ml)	Grapefruit juice	Commercial juice (pH 3.57, 8.05°Brix)	Continuous reactors with different configurations containing 1 or 2 lamps (24 cm × 2.5 cm) in a stainless steel tube (35 cm × 5 cm), peristaltic pump, and with/out ultrasonic atomizer nozzle	λ (253.7 nm), P (0.30-1.15 W), I (1.6-6.1 mW/cm^2^), flow rate (1.1 ml/s), 3 cycles (9.42 min), total dose (1.64-3.13 J/ml)	Ultrasonic atomizer nozzle	<0.5 log CFU/ml	The best treatment (<3 log CFU/ml)	[44]
	1 strain, initial load (6.0 log CFU/ml)	Tangerine juice	Commercial juice (pH 3.87, 10.10°Brix)				<0.5 log CFU/ml	The best treatment (<3 log CFU/ml)	
*Z. rouxii*	1 strain, initial load (6.0-7.0 log CFU/ml)	Apple juice	Commercial juice (pH 3.75, 10.1°Brix); 35 ml sample	Batch reactor with 64 LED lamps (22 × 22 cm), sample tray, and petridish (90 × 15 mm) for sample with stirrer	UV-C LED λ (275 nm), total dose (0.2-1.2 J/cm^2^), sample to lamp (15 cm)	NA	UV-C LED 1.2 J/cm^2^ (>5 log CFU/ml)	NA	[47]
*C. parapsilosis*	1 strain, initial load (4.0-5.0 log CFU/ml)	Orange juice	Commercial juice (pH 3.71, 11.60°Brix, insoluble solids 2.46%); 4 ml sample	Batch reactor with chamber, 3 lamps, and 35-mm petridish for sample with stirrer (1,500 rpm)	λ (254 nm), P (15 W), I (4.45 mW/cm^2^), dose (4.45 mJ/cm^2^ per s), exposure time (0-1,300 s), sample to lamp (10 cm)	NA	UV-C 0.6-0.7 J/cm^2^ (<5 log CFU/ml)	NA	[24]
*C. pseudointerme dia*	1 strain, initial load (4.0-5.0 log CFU/ml)						UV-C 2.5-3.0 J/cm^2^ (<5 log CFU/ml)		
*C. tropicalis*	1 strain, initial load (4.0-5.0 log CFU/ml)						UV-C 2.5-3.0 J/cm^2^ (<5 log CFU/ml)		
*Clavispora lusitanae*	1 strain, initial load (4.0-5.0 log CFU/ml)						UV-C 2.4-3.2 J/cm^2^ (<5 log CFU/ml)		
*Cryptococcus albidus*	1 strain, initial load (4.0-5.0 log CFU/ml)						UV-C 3.0-3.5 J/cm^2^ (<5 log CFU/ml)		
*Debaryomyces hansenii*	2 strains, initial load (4.0-5.0 log CFU/ml)						UV-C 1.2-1.4 J/cm^2^ (<5 log CFU/ml)		
*Kluyveromyces marxianus*	1 strain, initial load (4.0-5.0 log CFU/ml)						UV-C 1.0-1.2 J/cm^2^ (<5 log CFU/ml)		
*Meyerozyma guilliermondi*	1 strain, initial load (4.0-5.0 log CFU/ml)						UV-C 2.0-2.4 J/cm^2^ (<5 log CFU/ml)		
*P. anomala*	1 strain, initial load (4.0-5.0 log CFU/ml)						UV-C 1.0-1.2 J/cm^2^ (<5 log CFU/ml)		
*P. fermentans*	2 strains, initial load (4.0-5.0 log CFU/ml)						UV-C 1.0-2.0 J/cm^2^ (<5 log CFU/ml)		
*Torulaspora delbrueckii*	2 strains, initial load (4.0-5.0 log CFU/ml)						UV-C 1.2-1.4 J/cm^2^ (<5 log CFU/ml)		
*Trichosporon cutaeneum*	1 strain, initial load (4.0-5.0 log CFU/ml)						UV-C 3.6 J/cm^2^ (<5 log CFU/ml)		
*Composited yeasts*	17 strains, initial load (4.0-5.0 log CFU/ml)						UV-C 6.4-7.2 J/cm^2^ (<5 log CFU/ml)		
*Aspergillus fischeri*	Ascospores from 1 strain, initial load (6.0 log CFU/ml)	Apple juice	Commercial juice (pH 3.8, 12°Brix); 30 ml sample	Batch reactor with chamber (75 × 70 × 45 cm), 5 lamps, and petridish (4 mm depth) for sample	λ (254 nm), P (3 lamps with 15 W each; 2 lamps with 32 W each), I (6.5, 13, 21, 36 W/m^2^ 0-30 min), 30 min pre-irradiation, sample to lamp (20 cm)	NA	>5 log CFU/ml (UV-C 36 W/m^2^ 10 min)	NA	[32]
*Paecilomyces niveus*	Ascospores from 1 strain, initial load (5.0 log CFU/ml)						>4 log CFU/ml (UV-C 36 W/m^2^ 10 min)		
*Talaromyces macrosporus*	Ascospores from 1 strain, initial load (5.0-6.0 log CFU/ml)	Apple juice	Commercial juice (pH 3.83, UVA 12.56/cm); 30 ml sample	Batch reactor with 70 ml tank (76.5 cm × 1 mm) and heat controller	λ (254 nm), P (55 W), I (31 mW/cm^2^ 0-30 min), total dose (7.2, 14.3, 21.5 J/ml, 1-3 cycles)	Ultra-high pressure homogenization (100 or 200 MPa)	UV-C 21.5 J/ml (2 log CFU/ml)	UV-C 21.5 J/ml (>3 log CFU/ml)	[33]
*Neosartorya spinosa*	Ascospores from 1 strain, initial load (5.0-6.0 log CFU/ml)						UV-C 21.5 J/ml (>5 log CFU/ml)	UV-C 21.5 J/ml (>5 log CFU/ml)	

^a^°Brix (Soluble solids content); ρ (Density); UVT (UV transmittance); UVA (UV absorbance); NTU (Nephelometric turbidity units, NTU)

^b^ID (Inner diameter); OD (Outer diameter); DBD-driven KrCl excilamp (Dielectric barrier discharge-driven krypton-chlorine excimer lamp); LED (Light emitting diode)

^c^λ (Wavelength), P (Power), I (Irradiance/intensity); RT (Sample residence time)

^d^ChNPs (Chitosan nanoparticles); NA (Not applicable)

**Table 2:** Effects of ultraviolet C (UV-C) irradiation treatments on quality properties of different fruit juices.

Juice matrices	UV-C treatment procedure ^a^	Effects on quality properties ^b^	Reference
Apple juice	UV-C (222 nm; 0.17 mW/cm^2^ for 5-11 min; 45 and 55°C)	No significant changes were observed in the Color, TPC, and TAA (DPPH)	[25]
	UV LED (254-405 nm for 20-40 min)	A remarkable change in the color (a and b) was observed in the samples treated with a combined UV-C and UV-A LED	[45]
	UV-C LED (275 nm; total dose 0.2-1.2 J/cm^2^)	No significant changes in the pH, °Brix, acidity, and reducing sugar were observed (UV-C up to 1.2 J/cm^2^). Negative impacts on the TPC, TAA (DPPH, ABTS, and FRAP), and color parameters (a and b) were observed (UV-C ≥0.2 J/cm^2^)	[47]
Orange juice	UV-C (254 nm; 14 W/m^2^ for 3-15 min; total dose 2.52-12.6 kJ/m^2^)	Thiamine was preserved but vitamin C was not (UV-C at 12.6 kJ/m^2^)	[30]
	UV-C LED (279 nm; total dose 0.16-1.42 J/cm^2^)	No remarkable changes in the pH, °Brix, acidity, and color parameters (UV-C up to 1.42 J/cm^2^), but TPC significantly decreased (UV-C at ≥0.28 J/cm^2^)	[46]
Grape juice	UV-C (254 nm; 10-11 mW/cm^2^ for 60 min)	No significant changes in the °Brix and pH were observed. Significant changes in the color parameters (L, a , b) were observed, but they are not perceptible by the human eye	[36]
	UV-C (254 nm; 19.7 mW/cm^2^ for ≥20 min)	No significant changes were observed in the pH, acidity, °Brix, turbidity, and color. Significant loss of vitamin C (92%), TPC (19%), and TAA (54%) was observed	[40]
Carrot juice	UV-C (253.7 nm for 5 cycles; dose per cycle 1,152 J/L or 0.23 J/cm^2^)	No significant changes in the physicochemical (color, browning index, viscosity, optical density, density, pH, and turbidity) and sensory parameters were observed	[48]
Tomato juice	UV-C [(254 nm; 0.19 J/cm^2^ with ohmic heating (63°C, 210 s)]	Color and lycopene content were not significantly deteriorated by the combined treatment	[43]
Cranberry flavored water	UV-C [(254 nm; 94-376 s; 5-21 mJ/cm^2^ (doses when a 5 log reduction was achieved)]	No significant decrease on the concentration of anthocyanin and vitamin C was observed (UV-C at ≤30 mJ/cm^2^)	[19]
Grapefruit juice Tangerine juice	UV-C (253.7 nm; 1.6-6.1 mW/cm^2^ for 0-9.42 min; total dose 1.64-3.13 J/ml) combined with/out ultrasonic atomizer nozzle	No significant changes in the pH, °Brix, and color were observed	[44]
Pomegranate juice	UV-C [253 nm; 0.682 J/cm^2^ 45 min (individual) or 0.281 J/cm^2^ 20 min (combined ChNPs 100 μl/ml)]	UV-C alone: TPC was not affected but vitamin C and anthocyanin reduced. UV-C combined with ChNPs (100 μl/ml): TPC, vitamin C, and anthocyanin were not affected	[42]
Carrot-orange juice	UV-C (253.7 nm for 15 min; total dose 10.6 kJ/m^2^) combined with/out mild heat (50°C)	No significant changes in the pH, °Brix, and turbidity were observed. Changes in the TPC, TAA, and color were observed in the UVC-treated samples	[49]
Orange-tangerine juice	UV-C (253.7 nm for 0-15 min; total dose 0-1.72 J/cm^2^)	No significant changes were observed in the color, pH, acidity, °Brix, and turbidity (UV-C up to 1.72 J/cm^2^). TPC and TAA (DPPH) decreased significantly (UV-C at 1.72 J/cm^2^)	[28]

^a^LED (Light emitting diode); ChNPs (Chitosan nanoparticles)

^b^TPC (Total phenolic content); TAA [Total antioxidant activity; DPPH (DPPH free radicals scavenging activity assay), ABTS (ABTS Radical Scavenging Assay), FRAP (Ferric reducing antioxidant power assay)]; °Brix (Soluble solids content)
